# Biomarkers as Surrogates for Coronary Endothelial Dysfunction in Patients With Nonobstructive Coronary Artery Disease

**DOI:** 10.1161/JAHA.118.010166

**Published:** 2018-08-04

**Authors:** Anish Vani, Arthur Schwartzbard, Howard S. Weintraub

**Affiliations:** ^1^ Division of Cardiology New York University School of Medicine New York NY

**Keywords:** Editorials, biomarker, coronary artery disease, endothelium, prevention, risk factor, Endothelium/Vascular Type/Nitric Oxide

## Introduction

The endothelium is an organ system consisting of a dynamic cell layer with vital physiological functions.^1^ Endothelial dysfunction represents an amalgamation of traditional and nontraditional cardiovascular risk factors, genetic predisposition, local factors (eg, sheer), and yet undiscovered influences.^2^ Endothelial dysfunction can predispose to vascular remodeling, inflammation, vasoconstriction, thrombosis, and plaque rupture and erosion.^2^ There is prevailing evidence suggesting that epicardial and microvascular coronary endothelial dysfunction are strongly and independently associated with cardiovascular events in patients with or without coronary artery disease.^2^, ^3^, ^4^ Cardiovascular risk factor optimization has been shown to improve endothelial health and decrease future cardiovascular events.^2^, ^5^ The discovery of biomarkers as surrogates for endothelial dysfunction, therefore, could potentially serve to identify patients who are at greater risk for cardiovascular events and who may benefit from early and aggressive risk factor modification, including lifestyle changes, antiplatelet therapy, and statins.

Plasminogen activator inhibitor‐1 (PAI‐1) is an inhibitor of fibrinolysis with diurnal circadian variation, and higher serum PAI‐1 levels are associated with greater myocardial infarction risk.^6^, ^7^ Soluble urokinase plasminogen activator receptor (suPAR) is a proinflammatory marker with evidence suggesting it can outperform traditional markers of inflammation, such as C‐reactive protein, in prognosticating cardiovascular events.^8^ Whether these biomarkers can predict coronary endothelial dysfunction is an area of ongoing research.

In this issue of the *Journal of the American Heart Association* (*JAHA*), Corban and colleagues report their findings of 79 consecutive patients (mean age, 53±10 years) with angina who underwent clinically indicated coronary angiography and were found to have angiographically normal coronary arteries or mild epicardial coronary artery disease, defined as <40% stenosis.^9^ Patients with acute coronary syndrome, recent myocardial infarction or stroke, prior percutaneous coronary intervention, reduced ejection fraction, or other conditions (eg, active malignancy, infection, or pregnancy) were excluded. Study participants underwent coronary vasomotor testing with intracoronary adenosine to measure endothelium‐independent coronary flow reserve and with intracoronary acetylcholine to measure endothelium‐dependent coronary vasoreactivity. Epicardial endothelial dysfunction and microvascular endothelial dysfunction were defined as suboptimal responses to intracoronary acetylcholine infusion, as measured by percentage change in coronary artery diameter or coronary blood flow, respectively. Both systemic and local coronary blood was sampled for levels of PAI‐1 and suPAR before acetylcholine administration. Cross‐coronary left anterior descending artery biomarker production rates were calculated by subtracting the left main arterial and coronary sinus concentrations multiplied by the coronary blood flow.

After vasomotor testing, 28% of study participants were found to have endothelium‐independent dysfunction, 62% with microvascular endothelial dysfunction and 26% with epicardial endothelial dysfunction. Patients with microvascular endothelial dysfunction had higher rates of local coronary suPAR production compared with suPAR extraction in patients without microvascular endothelial dysfunction (median, 25.8 versus −12.7 ng/min; *P*=0.03), and patients with epicardial endothelial dysfunction had higher rates of local coronary PAI‐1 production compared with PAI‐1 extraction in patients without epicardial endothelial dysfunction (median, 1225 versus −187 ng/min; *P*=0.03). There were no significant differences in systemic (as measured by left main arterial blood) concentrations of suPAR, PAI‐1, or C‐reactive protein between groups. Local coronary suPAR and PAI‐1 production rates were not significantly different in patients with or without endothelium‐independent microvascular dysfunction.

In sum, this study by Corban and colleagues^9^ reveals that patients with endothelial dysfunction have increased cross‐coronary levels of suPAR or PAI‐1 production, whereas patients with normal endothelial function have suPAR and PAI‐1 extraction, providing a link between fibrinolytic and inflammatory pathways and endothelial dysfunction. Whether these biomarkers are causal or merely associated with coronary endothelial dysfunction warrants further investigation. Study limitations include a small sample size, a predominantly white population, the relatively invasive nature of obtaining data (ie, need for coronary catheterization with cross‐coronary blood sampling), and the absence of longitudinal and cardiovascular outcome data. It is not known if suPAR or PAI‐1 cross‐coronary production rates are affected by lifestyle changes and cardiovascular risk factor optimization.

Aggressive lipid‐lowering therapy and treatment with an anti‐inflammatory agent have been shown to reduce cardiovascular events in patients with elevated inflammatory markers in large randomized controlled trials,^10^, ^11^ with additional cardiovascular outcome trials for other agents lowering inflammation still ongoing.^12^ In a patient who does not fall into a traditional statin benefit group,^13^ further risk stratification can be useful for the clinician to decide how aggressively to manage a patient at moderate cardiovascular risk. This may be especially important in a patient who undergoes coronary angiography and is found to have zero or minimal coronary artery disease. Furthermore, in certain risk assessments, such as a preoperative evaluation, endothelial dysfunction has been associated with myocardial injury after noncardiac surgery.^14^ Additional data from the cardiac catheterization, such as the presence of elevated biomarkers associated with coronary endothelial dysfunction, may help to guide further management. The concept of endothelial dysfunction as a marker for cardiovascular risk and the use of biomarkers as surrogates for coronary endothelial dysfunction, while requiring further investigation before routine use in clinical practice, is certainly an area of intrigue in the quest to ultimately lower the burden of cardiovascular disease.

## Disclosures

Schwartzbard reports research support to New York University from Sanofi, Ionis, Merck, and Pfizer; he serves as an advisor to the formulary committee for Optum Rx. Weintraub reports research support from Amarin, Sanofi, and Ionis; Advisory: Amgen and Amarin. Vani has no disclosures to report.
